# Navigating Uncharted Territory: A Qualitative Analysis of Challenges and Advantages Experienced by Early Career Medical Educators

**DOI:** 10.1007/s40670-024-02205-7

**Published:** 2024-11-07

**Authors:** Caroline M. Gundler, Sara Allison

**Affiliations:** 1 Department of Biomedical Sciences, Ohio University Heritage College of Osteopathic Medicine, 6775 Bobcat Way, Dublin, OH 43016 USA; 2 Department of Biomedical Sciences, Western Michigan University Homer Stryker M.D. School of Medicine, Kalamazoo, MI USA

**Keywords:** Medical Educators, Early Career, Professional Development, Academic Climate

## Abstract

With the growing demand for medical educators, it is increasingly important that institutions implement practices to recruit, retain, and promote the well-being of early career medical educators (ECMEs). However, the specific needs of this group remain somewhat unclear. The current study aimed to address this gap by utilizing qualitative analysis to identify challenges and advantages associated with ECME status. An anonymous, voluntary survey was administered on forums of professional societies for medical education and on social media platforms, and emailed to professional societies. The survey collected demographic information and qualitative data regarding ECMEs’ experiences. Data were collected from 39 participants who met the ECME criteria of having less than or equal to 10 years of teaching experience since obtaining a terminal degree. Thematic analysis revealed that the challenges of early career faculty were difficulty balancing responsibilities, lack of research and educational support, perceived inadequacies as a faculty member, and development of workplace friendships. The advantages were the ability to understand students, possessing specific knowledge and skills, and the ability to innovate. ECME professional development needs focused on development of skills, networking, and support. In conclusion, ECMEs encounter similar struggles faced by all medical educators; however, they have the least amount of experience navigating the hurdles of academia. To aid ECMEs, institutions and departments can focus on the implementation of meaningful professional development opportunities, support systems, and encouraging ECMEs’ strengths.

**Supplementary Information:**

The online version contains supplementary material available at 10.1007/s40670-024-02205-7.

## Background

Medical educators play a critical role in the training of future physicians. They are responsible for shaping the environment in which medical students develop the knowledge and skills necessary to care for patients. Despite the significance of this career, the roles of a medical educator remain poorly defined. Several faculty identified as medical educators offered diverse definitions, ranging from a person who carries a passion for bringing out the best in learners to one that uses educational theories to guide their teaching, scholarship, and curricular design [1].

The lack of consensus around what it means to be a medical educator likely stems from the diverse and multifaceted nature of the role, which encompasses a wide range of responsibilities that differ significantly across the field [1, 2]. Some medical educators do most of their teaching in a traditional classroom setting, while others teach in laboratories or clinical environments. For some, teaching is at the very core of their career, while others balance significant patient care responsibilities or lead active research labs. These diverse roles result in a broad spectrum of responsibilities ranging from scholarship, policy development, program accreditation, faculty development, technical support, facilitation of learning events, student assessment, and providing social and emotional support for students [3]. In fact, Reisenburg and colleagues [4] identified as many as 237 potential job responsibilities associated with medical educator roles.

Others have attempted to establish core competencies for medical educators which include goals related to education, scholarship, administration, professionalism, reflective practice, content expertise, leadership, community engagement, mentoring, interpersonal skills, cultural competency, and navigating the medical education system [5, 6]. While already numerous, these responsibilities are rapidly evolving. An expert group at the Association of American Medical Colleges (AAMC) Learn, Serve, Lead conference formulated a list of roles that would be necessary for the success of a medical educator in 2025 [7]. The list suggests that the role will soon include utilization of big data to better understand individual student performance, early adoption of novel technological tools, and curation of content developed by national experts [7].

This ambiguity is compounded by the absence of a defined pathway to becoming a medical educator. Unlike more traditional career trajectories in research or clinical practice, the journey to becoming a medical educator lacks a clear roadmap. Therefore, many medical educators transition from clinical or research backgrounds [8]. To become a medical educator, some have pursued an advanced degree while others were mentored and observed others in the field. Some simply began to identify themselves as belonging to a community of experts while others made the leap simply by *doing* medical education [1]. Regardless of the chosen path, the transition is often marked by a sense of dissatisfaction with the support available [9], reflecting broader systemic issues in recognizing medical education as a legitimate career path [10].

For those able to make the transition into medical education, the pathway for success still remains somewhat ill-defined [10–12]. Many medical educators feel their work in the field is not recognized or is undervalued in comparison to research or clinical endeavors [2, 8, 11]. Others have cited many different measures of success for educators [13] making promotion in the field difficult [8] and leading to calls for established criteria for judging excellence in medical education [2].

The poorly outlined scope of activities in combination with the lack of common professional origin ultimately results in medical education lacking visibility as a career [3]. This has left many of those in the field struggling with professional identity formation [14]. Some faculty experience tension between what they feel is their primary role as a clinician or researcher and the role of medical educator [8, 11]. Brooks and colleagues [15] found that basic science medical educators experience specific threats to their professional identity formation such as struggling to balance conflicting roles, feeling untrained to teach, lacking meaningful clinical knowledge, and difficulty in forming an educator identity in an institution built to support research [15]. Sabel and colleagues summarized this struggle with identity formation, noting that “educators do not see themselves as belonging to the field of medical education. Instead they inhabit the field” (p. 1479) [11].

While medical educators at all stages face similar challenges, those early in their career are likely the most vulnerable given that they have the least amount of experience navigating the field. This group experiences the common challenges of balancing publishing, teaching, service, and life outside of work [16, 17], but they may not yet have the resources, experience, or network to effectively manage this balance. This has resulted in junior educators feeling isolated [18], overwhelmed [19], and emotionally exhausted [20]. One early career medical educator (ECME) specifically described their struggle to understand expectations and ask for help, which left them feeling overwhelmed and ultimately placed strain on their personal and professional relationships [21]. These negative experiences have the potential to lead to early career medical educator burnout which may affect institutions’ ability to retain and train high-quality medical educators [22, 23].

This is particularly troubling as concerns over physician shortages rise [24] and the demand for medical educators continues to increase [12, 25, 26]. Recognizing the pivotal role that early career medical educators will play in addressing the looming physician shortage, there is an urgent need to support these individuals and ensure they are retained. Factors such as lack of organizational support, low job satisfaction, difficulty balancing work and home life, higher stress, and burnout among those in higher education are associated with a higher likelihood of intention to quit [27]. In order to protect against these factors, there must be organized support for early career medical educators’ development and well-being.

While existing literature explores the challenges faced by medical educators at various career stages, there is a notable gap in understanding the unique difficulties encountered by early career professionals in this evolving field. Despite efforts to provide formal and informal developmental opportunities [1, 28–33], certain challenges such as teaching peers of a similar age and forming workplace relationships remain unexplored. Furthermore, no studies have investigated the potential benefits of early career status in medical education which may result in missed opportunities to capitalize on advantages and further inform strategies to support the growth and retention of early career faculty. This study addresses these gaps through qualitative analysis, aiming to provide a more nuanced understanding of both the challenges and advantages faced by early career medical educators, with implications for improving support and retention strategies.

## Materials and Methods

An Early Career Medical Educator Questionnaire (Appendix A) was created utilizing surveys from a literature search and was tailored towards medical educators [34–37]. Some of the items were directly adapted from existing validated surveys found in the literature (questions 11, 14–16). The decision process for selecting these items was based on their relevance and alignment with this study’s focus. Other questions were developed by the authors to address gaps identified in the existing literature and to capture aspects of the early career medical educator experience that were not previously explored. The questionnaire contained ten demographic questions regarding gender, age, ethnicity, teaching specialty, percentage of full-time equivalent (FTE) dedicated to teaching, type of degree, training focus, appointed teaching position, years teaching with terminal degree, and the student population they teach. This was followed by six open-ended questions concerning early career medical educators’ opinions about specific challenges and advantages experienced in their current role, their professional development needs, and where they seek professional advice.

The Early Career Medical Educator Questionnaire was an anonymous, voluntary survey administered via Qualtrics. Thirty-nine professional societies were identified through a Google search using keywords such as association, society, organization, allopathic, osteopathic, medical education, physiology, pharmacology, pathology, microbiology, histology, immunology, neuroscience, and anatomy. Based on this search, the survey was posted on forums of professional societies for medical education that allowed access to posting on their forum (American Association for Anatomy; American Association of Colleges of Osteopathic Medicine; Association for Medical Education in Europe) and social media outlets (Twitter/X, LinkedIn, Facebook). The survey link and posting were publicly available, and all individuals were encouraged to share them widely with their networks. To increase exposure of the survey, it was also emailed to 22 professional societies inquiring if they would share the survey with their members. The professional societies emailed were the following: Association of American Medical Colleges (AAMC) IPE MedEd Portal; Association for Medical Education in Europe; Foundation for Advancement of International Medical Education and Research; Advancing Scholarship in Medical Education (ASME); World Federation for Medical Education (WFME); Canadian Association for Medical Education (CAME); Gesellschaft fur Medizinische Ausbildung (GMA); Academy of Medical Educators (AoME); Scientific Medical Society of Anatomists, Histologists and Embryologists; Anatomical Society; Society for Neuroscience; American Neurological Association; International Society of Neuroscience; American Association of Immunologists; The Physiological Society; American Society for Matrix Biology (ASMB); Society for Experimental Biology and Medicine (SEBM); Society of Ultrasound in Medical Education (SUSME); American Society for Clinical Pathology; International Union of Basic and Clinical Pharmacology (IUPHAR); American Society for Pharmacology and Experimental Therapeutics (ASPET); and International Association of Medical Science Educators (IAMSE). Because of the anonymous nature of the survey, correlations between survey participation and society connection could not be made. The survey was reposted 1 month after the original posting and was open for 2 months between February 2022 and April 2022.

In alignment with previous literature, an early career medical educator was defined as having less than or equal to 10 years of teaching experience since obtaining a terminal degree [37]. Any survey respondents that did not meet this criterion were removed from data analysis. It is important to note that this definition encompasses individuals who may be over 40 years of age or hold associate or full professor appointments. These individuals were included if they had 10 years or less of teaching experience after obtaining their terminal degrees, as many may have transitioned from other professional careers into academic teaching roles later in their careers.

The qualitative data were analyzed using thematic analysis, which was conducted by the authors without the use of qualitative data analysis software. Thematic analysis involves analysis of the free-text responses and development of patterns or themes from this analysis. First, codes were produced from the free responses to create potential themes or sub-themes. The sub-themes were then reviewed and collated to create main themes [38]. For the demographic information, data were analyzed using descriptive statistics. To maintain the anonymity of the participants, no identifiers were collected. The study was deemed exempt by Ohio University’s Institutional Review Board (IRB# 21-E-405).

## Results

### Demographic Information

**Fig. 1:**
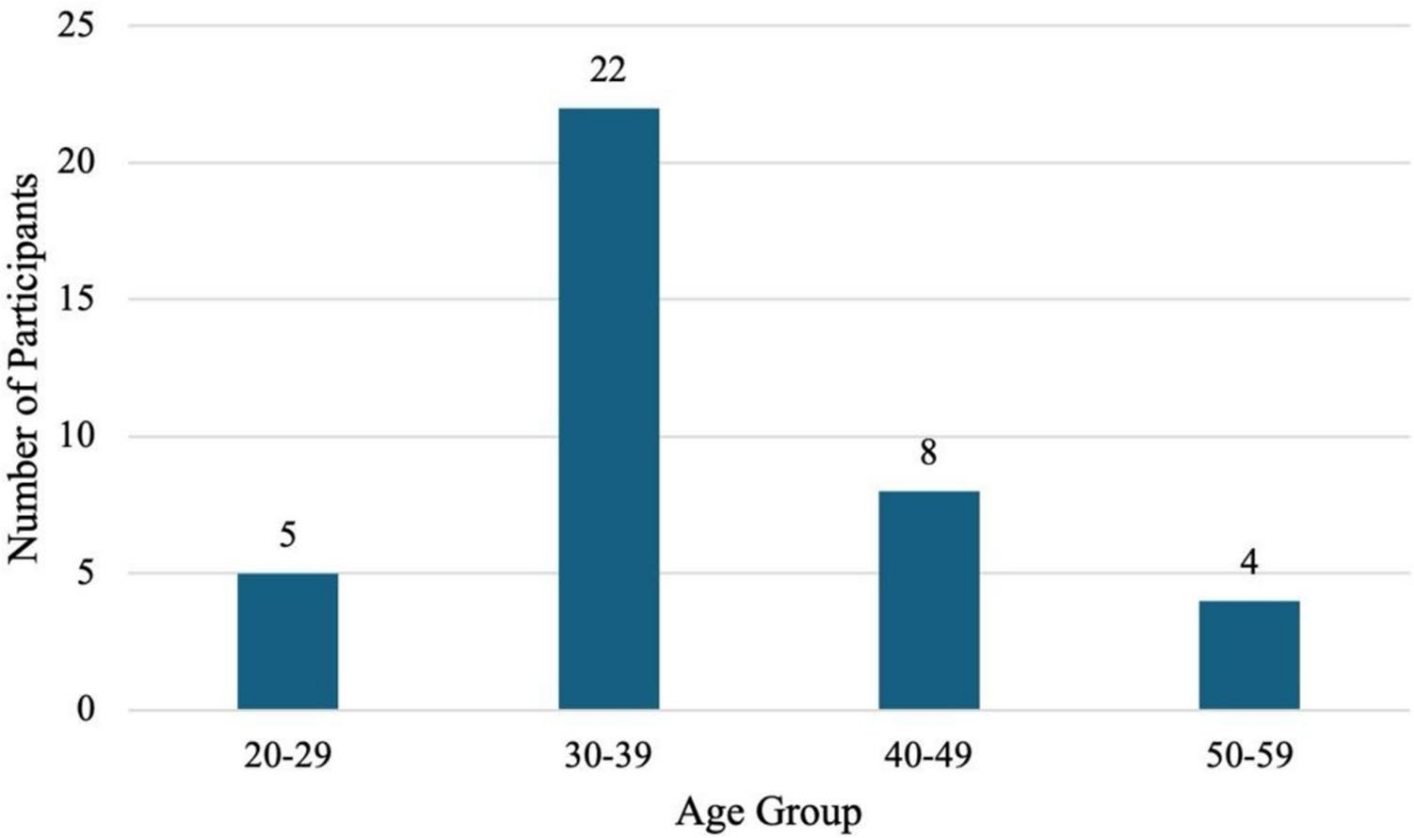
Age of early career medical educators

Forty-nine responses were collected, with 39 individuals qualifying as early career medical educators (ECME). Of these participants, 71.8% were female (*n* = 28), 25.6% were male (*n* = 10), and 2.6% reported other (*n* = 1). The majority of the participants self-identified as White (79.5%, *n* = 31), 5.1% as African American/Black (*n* = 2), 2.6% as Asian (*n* = 1), 2.6% as Hispanic/Latino (*n* = 1), 2.6% preferred not to answer (*n* = 1), and 7.7% selected Other (*n* = 3). Those that selected “Other” self-identified as mixed; White and Chinese; and Turkish. The average age of the participants was 37 years old (Fig. 1).

**Fig. 2:**
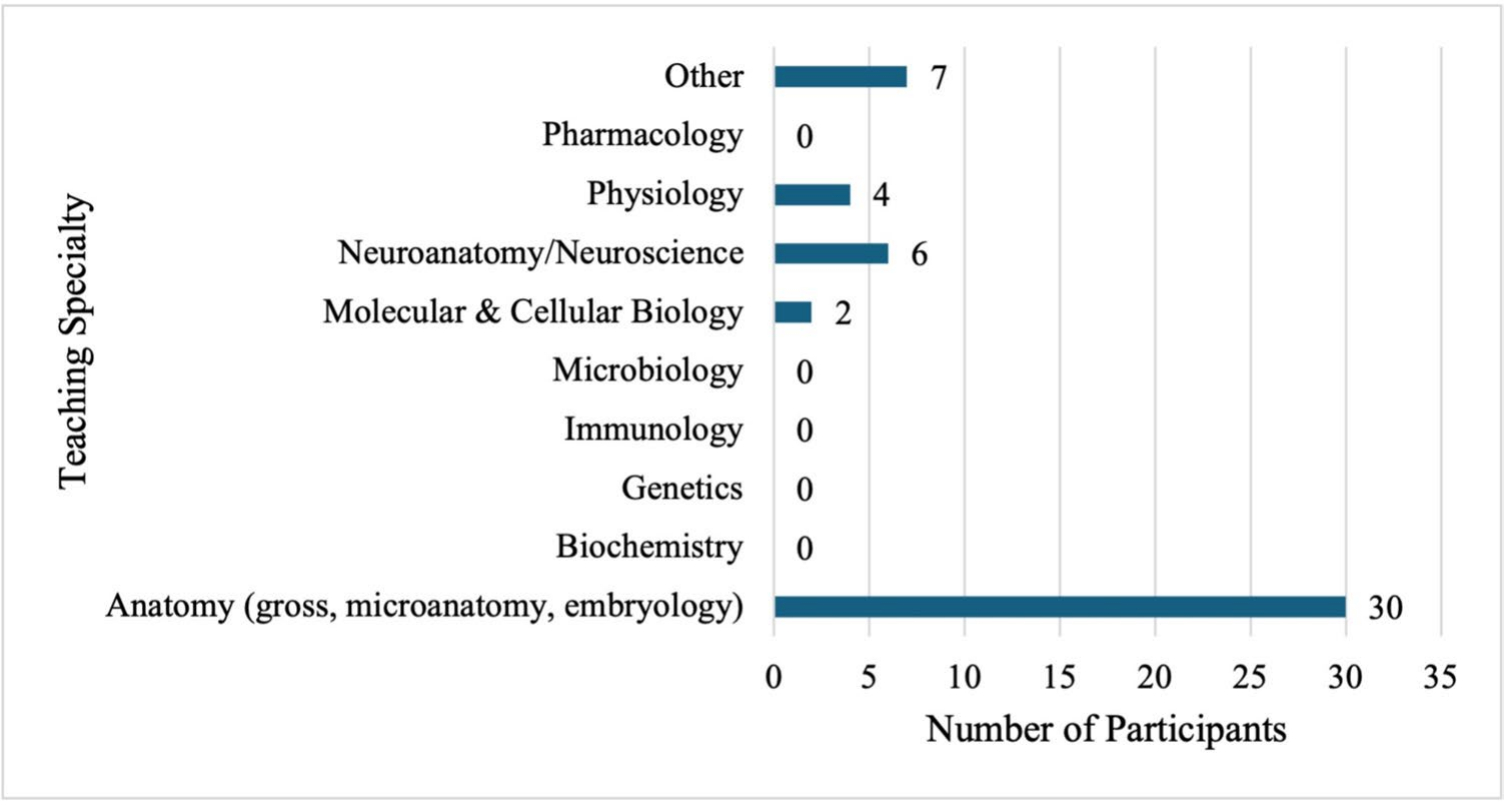
Teaching specialty. Note that the 39 study participants were able to indicate more than one teaching specialty area

Anatomy was the main discipline taught by the early career educators (61.2%, *n* = 30) (Fig. 2). Those that selected “Other” for their teaching specialty listed the following: family medicine; public health sciences; medical education; pathology and histology; and pathology. Two individuals who selected “Other” left no response. Seventeen participants (43.6%) indicated that their training was research-based, 18 indicated it was educational (46.2%), two indicated clinical (5.1%), and two indicated both educational and research-based (5.1%). The average percentage of FTE dedicated to teaching was 61% (*n* = 36) with three individuals not reporting their FTE. Table 1 summarizes the degrees held by participants and their appointed teaching position. ECMEs’ average years teaching with their degree was 4.05 years. ECMEs taught medical students (47.8%, *n* = 33), Master’s students (15.9%, *n* = 11), doctoral students (14.5%, *n* = 10), undergraduate students (11.6%, *n* = 8), and residents (10.1%, *n* = 7).

**Table 1:** Degrees and appointed teaching positions held by early career medical educators

Demographic	Frequency (*n*)	Percentage (%)
Degree		
PhD	27	69.2
PhD, Master’s	3	7.7
PhD, DVM	1	2.6
PhD, MD	1	2.6
MD	2	5.1
Master’s	3	7.7
EdD	1	2.6
BS, BA	1	2.6
Appointed teaching positions		
Instructor	6	15.4
Assistant professor	24	61.5
Associate professor	6	15.4
Full professor	1	2.6
Other	2	5.1

### Qualitative Analysis

Thematic analysis was used to assess all six open-ended questions to delineate major themes from open-ended responses. The six questions are subdivided into three categories: challenges, advantages, and professional development. The first three questions ask ECMEs about the challenges and advantages of (1) being an early career professional, (2) teaching students in a similar age range, and (3) developing workplace friendships as an ECME.

Thematic analysis revealed that ECME’s overall challenges were difficulty balancing responsibilities; lack of research and educational support; and perceived inadequacies as a faculty member. Overall advantages of being an ECME included the ability to understand students, possessing specific knowledge and skills, and the ability to innovate.

#### Difficulty Balancing Responsibilities

ECMEs felt that a challenge was “balancing teaching, research, and service and making them compatible with family life (i.e., work-life balance).” Entering a new institution, learning the techniques of that institution, starting new courses, and adjusting to life as a faculty member contribute to difficulties establishing an optimal work-life balance.

#### Lack of Research and Educational Support

Another theme delineated from this question was lack of research and educational support as an ECME. This theme encompassed the low FTE/time/resources for research opportunities combined with a heavy teaching load, as well as a lack of clearly defined educational expectations. Participants stated that “there is no protected time for research associated with my position, so I am struggling.” Additionally, they felt a “lack of support when implementing pedagogy” and a lack of “clear expectations on how to […] spend [their] time.”

#### Perceived Inadequacies as a Faculty Member

This theme was multifaceted as it stemmed from ECMEs’ perceptions of how they were viewed by students and senior faculty as well as ECMEs’ perceptions of themselves. ECMEs claimed that students compared them to other faculty members who had more experience and/or faculty with a medical degree. Additionally, they felt that they were working to gain their colleagues’ approval by proving their knowledge and striving to be seen “as a peer to senior faculty, rather than a student/postdoc.” Some participants also struggled with imposter syndrome and had to work to convince themselves that they were worthy of their position.

#### Ability to Understand Students

Since ECMEs are more likely to have recently graduated from higher education, they felt that they understood more of the challenges of their students. This made faculty more approachable because they could relate to their students. One participant stated that “I still understand what my students are going through from a professional and personal level, because I am not that far removed from it myself. It helps me better understand their struggles.”

#### Possessing Specific Knowledge and Skills

This theme came from sub-themes related to ECME’s skills and knowledge of technology and modern resources such as up-to-date study resources, pedagogical skills, and presentation skills. ECMEs believe that they are “‘in touch’ with current research technology” and have the “methods of higher education and pedagogy and assessment.”

#### Ability to Innovate

As fresh eyes entering a new institution, ECMEs “can bring fresh and creative ideas into my teaching because I haven’t been doing it for a long time. I don't feel tied to a certain way of doing things.” Additionally, many ECMEs recently completed their educational training and maintain a relative proximity to the learning process which can provide a unique perspective on potential ways to present material.

The themes related to challenges and advantages of teaching students that are of similar age and developing workplace friendships are outlined in Table 2.

**Table 2:** Themes that emerged from questions about teaching students similar in age and developing workplace friendships

Themes	Descriptions	Exemplar quotes
**Q2: What are the challenges and advantages of teaching students that are similar in age to you?**		
*Challenges*		
Lack of student trust	Many ECMEs expressed that they felt questioned by students about their experience and accuracy of their knowledge	“I felt my expertise was also challenged more often than that of more senior faculty—students would more often debate facts or the accuracy of exam questions with me.”
Difficulty maintaining professional distance	ECMEs reported that students made inappropriate social requests and sometimes felt an undue level of connection due to their similar ages	“There is a difficult balance of being approachable to students without having students slip into being a bit too familiar and too casual.”
Unequal treatment based on position	Students and faculty would mistake ECMEs as students, assuming inexperience and a lesser level of authority. They felt that they often were treated as a student	“Sometimes I get mistaken as a student so this causes some students (and faculty) to treat me with less authority than some of my older colleagues.”
*Advantages*		
More approachable to students	ECMEs felt they were more approachable to students because they could connect with their life circumstances and were not far-removed from the material	“Younger age has the advantage of hopefully making students less intimidated by me and more likely to ask questions and come to office hours.”
Facilitated a connection with students	Similar life stages and experiences enabled natural connections between ECMEs and students	“I feel I have an easier time reading my students and building a relationship with my students than my older colleagues.”
**Q3: What are the challenges and benefits of developing workplace friendships as an early career professor?**		
*Challenges*		
Difficult due to age differences	Many ECMEs felt it was hard to connect personally and professionally since many faculty were at different stages of life	“The closest faculty in age is about 25 years older than me. It is difficult not having faculty members close in age and life stage (growing family).”
Takes time to form meaningful relationships	ECMEs relayed that it takes time to connect with other faculty and to prioritize doing things outside of work to foster workplace relationships	“Meeting new people and feeling comfortable with them takes time. The transition to becoming faculty can be jarring, full of imposter syndrome and lots of new experiences you don't know how to navigate.”
Challenges to connect because of COVID	During and after COVID, workplace connections were hindered since many people take meetings from home and/or do not set times to meet up outside of work	“Making workplace friendships is difficult right now due to lack of face to face meetings in the COVID era.”
*Advantages*		
Support system	ECMEs highlighted that workplace friendships provide them with support on the daily tasks to life advice	“Having others to count on for support.”
Enjoyable work environment	Many participants stated that having workplace friendships enhanced their work environment and happiness	“The benefits of developing work friendships is that work friends provide comfort and support. Having work friends just makes you feel good.”
To help advance career	ECMEs felt that having a workplace friendship gave them great advice whether it be personal or professional	“It's great to have near-peer colleagues to help navigate the bureaucracy (and endless acronyms) of a new institution. It's also good to have someone to vent to that is going through the same things or operating under the same conditions.”

### Professional Development

Concerning professional development, three topics were examined: (1) where ECMEs seek professional advice; (2) type of professional activities most important to ECMEs; and (3) ECMEs’ opinion on if they are offered adequate professional development opportunities. ECMEs seek professional advice from senior colleagues, early career faculty, mid-career faculty, professional society members, and graduate advisors and colleagues. Some ECMEs seek advice from multiple different individuals because who they “seek advice from is dependent on the issue at hand.” Other ECMEs do not approach individuals at their institutions “because there are no early career colleagues, and probably no mid-career colleagues at [their] college.” Essentially, they are limited at their institutions and therefore seek outside support for professional advice.

When asked if ECMEs have adequate opportunities for professional development, three main themes were delineated from the open-ended responses: (1) lack of funding and protected time; (2) limited or inadequate offerings; (3) adequate opportunities and support.

#### Lack of Funding and Protected Time

ECMEs described that there was “very little funding and very little protected time” for professional development activities. They claimed that it was difficult to prioritize professional development when teaching, doing research, completing service requirements, and attending to other components of their FTE that take higher priority. Additionally, “there are not enough professional development funds” causing ECMEs to go “over [their] budgets.”

#### Limited or Inadequate Offerings

Some ECMEs felt that they have “few resources offered by [their] universities.” They stated that even if there are resources or opportunities, sometimes they do not materialize, or they are not supported by the institution. Some participants stated that even though “there is adequate time and funds, the problem is the lack of offerings.”

#### Adequate Opportunities and Support

Other ECMEs stated that they have “adequate opportunities for professional development.” Participants stated that this level of support stems from overall institutional support for professional development from chairs, mentors, and academic deans. Some ECMEs stated that they do receive adequate time and funding; however, they “had to explore resources for PD on their own time.”

Concerning the types of professional activities ECMEs stated were most important to them, five areas were addressed: (1) development of pedagogical skills; (2) leadership and service opportunities; (3) development of research skills; (4) assistance with promotion; and (5) networking and conference presentations. Exemplar quotes for these themes are outlined in Table 3.

**Table 3:** Themes that emerged from what type of professional development activities are most important to ECMEs

Themes	Exemplar quotes
Development of pedagogical skills	• “How to advance my career in teaching strategies” • “Student engagement brought on by the pandemic and the permanent changes it is causing, and larger curriculum design”
Leadership and service opportunities	• “What is needed is opportunities to have more of a role in administration or leadership” • “Learning how to manage people and do the administrative work”
Development of research skills	• “Collaborate on multiple research projects, and how to manage multiple projects” • “Grant writing training, laboratory management”
Assistance with promotion	• “Potential timelines for promotion” • “A committee or team to help understand promotion and tenure”
Networking and conference presentations	• “Networking and presenting at national and international meetings” • “Figuring out ways to network”

## Discussion

This study highlights important gaps in the existing literature by focusing on the unique challenges and potential benefits experienced by early career medical educators (ECMEs). While much of the previous research has addressed obstacles faced by medical educators at various career stages, specific early career issues such as teaching peers of a similar age and forming workplace relationships have largely been overlooked. Additionally, the absence of studies examining the advantages of early career status suggests missed opportunities to leverage these benefits for professional growth and retention. By utilizing qualitative analysis, this study offers new insights into both the difficulties and advantages early career educators encounter, providing valuable implications for future support and development strategies.

### Difficulty Balancing Responsibilities

Early career medical educators in this study specifically highlighted their struggle to balance their new scholarship, teaching, and service responsibilities with their personal life. While this challenge may not be unique to ECMEs [16, 17], this population is less likely to have acquired the experience, skills, and tools to properly manage these increased demands. The significant ambiguity surrounding the roles of medical educators [3, 4] and lack of clearly defined pathway for success likely contribute to ECME’s difficulty in establishing a satisfactory balance. Instead of appropriately prioritizing and accomplishing tasks, ECMEs are spending precious time struggling to understand expectations. This ultimately exacerbates the stress inherently associated with navigating new workplace dynamics and likely contributes to the higher risk of burnout among early career educators [39].

To help ECMEs establish balance between competing responsibilities, institutions should strongly consider taking the time to establish and provide detailed and clearly defined expectations [28]. This helps ECMEs fully understand their duties, facilitates prioritization of tasks that will lead to individual and institutional success, and may reduce ECME’s feelings of being overwhelmed and burnt out [28]. It may also be beneficial for institutions to reinforce time management techniques (e.g., blocking off time for research, using planners, breaking down large goals into smaller ones) to improve the productivity and well-being of educators [40]. Establishing a network of adequate mentors that support ECMEs has also been cited as a critical component of faculty development [1, 16, 18, 29, 31]. Mentors may vary in their levels of expertise regarding pedagogy, content knowledge, and scholarship skills and ECMEs will likely require more than one mentor to accommodate their individualized needs. Mentors that share similar experiences and have overcome similar huddles are able to share tips for success and guide ECMEs on how to navigate and balance their new responsibilities.

### Lack of Support

Early career medical educators in this study also reported a lack of research and educational support. Institutions may consider protecting ECME’s time at the beginning of their career as designing teaching materials may take longer for novices who are still working to develop pedagogical skills and become an expert in their discipline. Similarly, ECMEs will likely require more time to complete scholarship-related tasks. Institutions and departments will ultimately benefit if they allow ECMEs the time and space to develop these critical skills.

Early career medical educators should also be encouraged to participate in focused professional development opportunities in the form of seminars, conferences, retreats, online webinars, and workshops. Several organizations have successfully provided training and certificate programs specifically tailored for medical educators [41–43]. These opportunities highlight the need for adequate funding for ECME development, a sentiment echoed by the participants of the current study. Standardized funding should be prioritized, eliminating the need for faculty to repeatedly request funds, and instead providing a consistent amount every year dedicated to professional development. In addition to funded opportunities, institutions can consider exploring no-cost opportunities for skill development. For example, peer observation of teaching sessions can provide an avenue for constructive feedback for ECMEs about teaching practices in a non-threatening way [44].

While many ECMEs in the current study focused on the lack of research and educational support, the specific professional development needs of each individual are highly variable. Table 3 provides an overview of the needs highlighted by the participants of this study. Professional development that is tailored to ECMEs can help them achieve their goals and increase their overall well-being which may ultimately help institutions retain faculty members.

### Perceived Inadequacies

Early career medical educators also expressed concern about perceived inadequacies as a faculty member. This theme stemmed from ECMEs’ perception of themselves and their perception of how other faculty and students viewed them. Some ECMEs felt that they were seen by senior faculty and students “as a student so this causes some students (and faculty) to treat [them] with less authority than some of [their] older colleagues.” The average age of medical students is in their mid–late 20 s [45], while many ECMEs are in their 30 s. This may discourage other faculty from interacting with ECMEs as peers or lead them to undervalue ECMEs opinions due to a perceived lack of experience. This proximity to student age may also contribute to students’ lack of trust in ECME’s ability to comprehend and teach the content compared to senior faculty. One ECME found that a student’s “perceived closeness to me as a peer, rather than an authority figure, can sometimes lead to a loss of professionalism” which can result in inappropriate social requests. Mentoring ECMEs on how to handle unusual situations with both students and faculty is critical. Connecting ECMEs with other early career educators to encourage peer mentoring can provide additional emotional and professional support [46]. Such mentoring can help ECMEs maintain a professional distance from students and establish their authority within the academic environment.

Some ECME’s perceptions of themselves contributed to their feelings of inadequacy. They reported feeling “unprepared or incapable” and questioned their own qualifications to be in their roles. Their perceptions of themselves may stem from imposter syndrome, which is the feeling that they are incompetent or undeserving to be in their position or role [47]. Encouraging ECMEs to discuss their feelings with support systems can help cope with imposter syndrome [48]. Having structured or informal sessions with experienced faculty can demonstrate that most faculty, both ECMEs and experienced faculty, have struggled or currently struggle with imposter syndrome and similar challenges. Additionally, resources such as workshops [49] and educational sessions involving small-group work [50] have been reported to combat imposter syndrome.

### Workplace Friendships

**Fig. 3:**
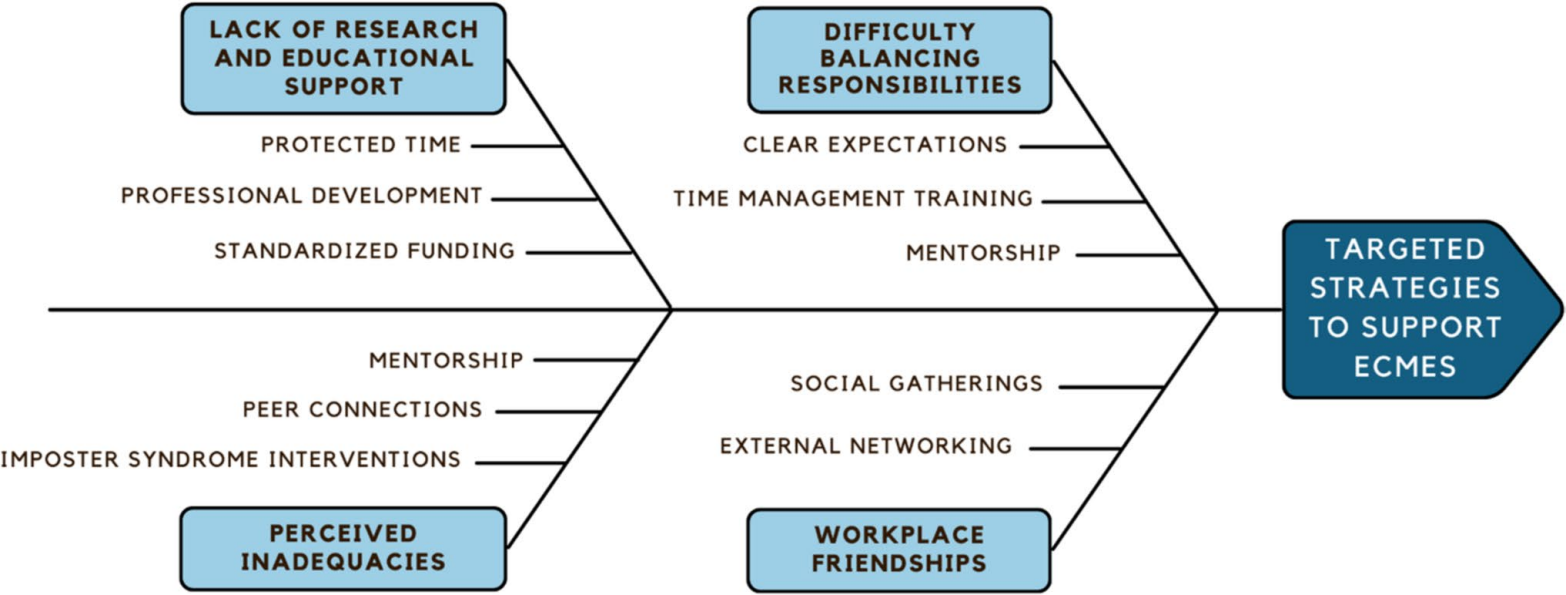
Targeted strategies to address challenges experienced by early career medical educators

Participants of this study identified some of the benefits of workplace friendships. As established by previous studies, the connections provided by workplace friendships are helpful in emotional, instrumental (e.g., tangible aid), and informational support [51]. Additionally, workplace friendships can provide a safeguard for work-related stress, aid against dissatisfaction in the workplace [51], and increase overall workplace happiness [52]. However, the participants of the current study cited age differences, time requirements, and COVID practices as barriers to creating these relationships. Institutions may consider establishing social outings, work lunches, and team building events to get ECMEs connected to other faculty and increase workplace rapport. Institutions should also prioritize connecting their ECMEs with ECMEs at other institutions, particularly if there are few ECMEs at their institution. Creating an internal and external network among ECMEs provides connections to those with similar experiences, helps ground the ECMEs in the field of medical education, provides a sense of belonging, and may promote retention. Figure 3 summarizes the most significant challenges identified by this study and highlights potential interventions.

### Early Career Medical Educator Advantages

Although there are various challenges for ECMEs, they also enjoy some specific advantages. Because many ECMEs completed their schooling relatively recently, they have a unique ability to understand and relate to students. Their proximity in age and shared experiences may help them appear more approachable and allow them to form unique connections with students. The importance of these teacher-student relationships should not be overlooked as they are often cited as the most rewarding aspect of a career in medical education [15]. ECMEs’ proximity to learning the content may also mean that they are able to utilize a shared language with students that allows them to explain complex topics at a more appropriate level.

They also bring a fresh perspective to curriculum development and quality improvement projects. They have immense potential to design innovative and technology-driven approaches to content delivery. Identification and celebration of these strengths are essential. Recognizing the value, they add to the medical education team which may help boost ECMEs’ confidence and moderate feelings of imposter syndrome. By capitalizing on individual ECME strengths and supporting opportunities for professional development, institutions can support their goals of recruiting and retaining medical educators to support the next generation of healthcare providers.

## Limitations

The current study has some limitations. The participants of this study represent a single subset of the early career medical educators throughout the USA. While there is no source which provides complete data on all early career medical educators as defined by this study’s criteria, the 2023 AAMC U.S. Medical School Faculty Report [53] provides demographic information for basic science educators at the assistant professor level which can be used for comparison. According to this report [53], the average age of assistant professors was 44.8, 56.7% identified as White, and 51.3% identified as female. For comparison, the average age of participants in the current study was 37, 79.5% self-identified as White, and 71.8% self-identified as female. Additionally, a majority of respondents were anatomists. This may be due, in part, to the multitude of anatomy programs that are tailored for careers in medical education [54]. Therefore, generalizability to ECMEs who have different identities or areas of expertise than those represented in the current study may be limited as these factors likely play a significant role in an individual’s experience of the workplace.

The authors also recognize that those who participated in the survey were most likely to hold strong positive or negative opinions related to their early career medical educator journey. It is likely that the general population of early career medical educator experiences fall somewhere along the spectrum of challenges and advantages outlined by these participants. Finally, at the time of this study, both researchers were ECMEs. The researchers acknowledge that their unique perspectives have informed this work and employed reflexivity to reflect upon their own assumptions, beliefs, and judgments.

## Future Directions

Future studies may focus on the development of ECME professional development activities which could be implemented upon starting a medical educator role or as courses in doctoral/postdoctoral programs prior to ECMEs entering the workforce. As indicated in the limitations, the authors recognize that there are a multitude of personal identities, demographic data, and job-specific responsibilities (e.g., the level of trainee being taught, the specific medical school mission) which will almost certainly influence the specific challenges and advantages experienced by early career medical educators. While the current study captures the general experiences of a subset of ECMEs, future studies may build upon this work by exploring the unique difficulties associated with specific populations of ECMEs. This may be especially important to consider among populations that are underrepresented in medicine as these groups may be particularly vulnerable to burnout [23]. Additionally, the study can be expanded by increasing survey outreach to include more disciplines and conducting faculty member interviews to further explore the difficulties and advantages associated with ECME status. Future studies may also build upon this research through the inclusion of researchers from different career stages.

## Conclusion

Overall, ECMEs experience similar struggles faced by all medical educators, such as difficulty balancing responsibilities and lack of research and educational support. However, they have the least amount of experience navigating these traditional hurdles of academia. They also encounter unique challenges related to perceived inadequacies and difficulties associated with establishing workplace friendships. To aid ECMEs, institutions and departments can focus on helping ECMEs create meaningful support systems (e.g., mentorships, workplace friendships), creating clear expectations, and emphasizing professional development programs early in their career. Additionally, institutions should celebrate and capitalize upon ECMEs’ strengths by encouraging their creativity, innovation, and ability to connect with students.

## Supplementary Information

Below is the link to the electronic supplementary material.

Supplementary file1 (DOCX 19 KB)

## Data Availability

The datasets generated during and/or analyzed during the current study are available from the corresponding author on reasonable request.
