# Supplementary material for: Navigating Uncharted Territory: A Qualitative Analysis of Challenges and Advantages Experienced by Early Career Medical Educators
Source: Med Sci Educ. 2024 Nov 7;35(1):403–14. doi: 10.1007/s40670-024-02205-7 (PMC11933481; doi:10.1007/s40670-024-02205-7)
Supplement: Supplementary file 1 — Supplementary file1 (DOCX 19 KB) [file 40670_2024_2205_MOESM1_ESM.docx]

**Appendix A: Early Career Medical Educator Questionnaire**

The following questionnaire is assessing the climate of early career medical educators. Please answer to the best of your ability. All responses will remain anonymous.

**Part 1: Demographics**

In the following table, either check the box(es) that applies or write in the appropriate response.

| 1. Sex:  - Male - Female - Prefer not to specify - Other: _______________ | 1. Age: ________________ |
| --- | --- |
| 1. Ethnicity:  - American Indian or Alaska Native - Asian - African American/Black - Hispanic/Latino - Native Hawaiian or Other Pacific Islander - White - Prefer not to say - Other: ________________________ | 1. Specialty:  - Anatomy (gross anatomy, microanatomy, embryology) - Biochemistry - Genetics - Immunology - Microbiology - Molecular & Cellular Biology - Neuroanatomy/Neuroscience - Physiology - Pharmacology - Other: _______________________ |
| 1. Percentage of FTE Teaching: ___________ | 1. Type of Degree:  - PhD - MD - DO - Master's - Other: _________________________ |
| 1. Training:  - Research-based (e.g., bench research) - Educational - Clinical - Other: ________________________ | 1. Appointed Position:  - Instructor - Assistant Professor - Associate Professor - Full Professor - Other: _________________________ |
| 1. Years Teaching with Terminal Degree: ____________________________________ | 1. Educational Level You Teach:  - Undergraduate students - Master's students - Doctoral students - Medical students (M.D. or D.O.) - Residents |

**Part 2: Open-Ended Questions**

1. What is your number one challenge and number one advantage that you experience as an early career professional?
2. What are the challenges and advantages of teaching students that are similar in age to you?
3. What are the challenges and benefits of developing workplace friendships as an early career professor?
4. Do you feel you are offered adequate opportunities for professional development (funds, protected time, institutional developmental offerings)? Please explain.
5. What type of professional development activities are most important to you at this stage in your career?
6. Who are you most likely to seek professional advice from (ex. senior colleagues, mid-career colleagues, other early career colleagues, professional society members, other)?
